# Macrophage Lamin A/C Regulates Inflammation and the Development of Obesity-Induced Insulin Resistance

**DOI:** 10.3389/fimmu.2018.00696

**Published:** 2018-04-20

**Authors:** Youngjo Kim, Princess Wendy Bayona, Miri Kim, Jiyeon Chang, Sunmin Hong, Yoona Park, Andrea Budiman, Yong-Jin Kim, Chang Yong Choi, Woo Seok Kim, Jongsoon Lee, Kae Won Cho

**Affiliations:** ^1^Soonchunhyang Institute of Medi-Bio Science (SIMS), Soonchunhyang University, Cheon-an, South Korea; ^2^Department of Surgery, Soonchunhyang University Hospital, Seoul, South Korea; ^3^Department of Plastic and Reconstructive Surgery, Soonchunhyang University Hospital, Gumi, South Korea; ^4^Department of Surgery, Soonchunhyang University Gumi Hospital, Gumi, South Korea; ^5^The Joslin Diabetes Center, Department of Medicine, Harvard Medical School, Boston, MA, United States

**Keywords:** lamin A/C, obesity, inflammation, insulin resistance, macrophages, adipose tissue

## Abstract

Obesity-induced chronic low-grade inflammation, in particular in adipose tissue, contributes to the development of insulin resistance and type 2 diabetes. However, the mechanism by which obesity induces adipose tissue inflammation has not been completely elucidated. Recent studies suggest that alteration of the nuclear lamina is associated with age-associated chronic inflammation in humans and fly. These findings led us to investigate whether the nuclear lamina regulates obesity-mediated chronic inflammation. In this study, we show that lamin A/C mediates inflammation in macrophages. The gene and protein expression levels of lamin A/C are significantly increased in epididymal adipose tissues from obese rodent models and omental fat from obese human subjects compared to their lean controls. Flow cytometry and gene expression analyses reveal that the protein and gene expression levels of lamin A/C are increased in adipose tissue macrophages (ATMs) by obesity. We further show that ectopic overexpression of lamin A/C in macrophages spontaneously activates NF-κB, and increases the gene expression levels of proinflammatory genes, such as *Il6, Tnf, Ccl2*, and *Nos2*. Conversely, deletion of lamin A/C in macrophages reduces LPS-induced expression of these proinflammatory genes. Importantly, we find that myeloid cell-specific lamin A/C deficiency ameliorates obesity-induced insulin resistance and adipose tissue inflammation. Thus, our data suggest that lamin A/C mediates the activation of ATM inflammation by regulating NF-κB, thereby contributing to the development of obesity-induced insulin resistance.

## Introduction

It has been well established that obesity-induced low-grade chronic inflammation contributes to the development of insulin resistance and type 2 diabetes in obesity. It has also been shown that white adipose tissue is the primary site for obesity-induced inflammation (1, 2), which is largely regulated by the quantitative and qualitative alterations of adipose tissue leukocytes (3–6). Adipose tissue macrophages (ATMs) are the most abundant cell types among adipose tissue leukocytes and also considered as final effector cells to regulate adipose tissue inflammation (2, 7). ATMs are categorized as either an M1 or M2 subset, which is well established in the classical immunology field, and the polarization of M1 and M2 macrophage phenotypes is switched by obesity (4, 5). Furthermore, M1 and M2 ATM phenotypes play a critical role in the regulation of obesity-induced inflammation and insulin resistance. In lean adipose tissue, anti-inflammatory M2 macrophages (M2 ATMs) are predominant (4, 8). During obesity, another type of ATMs demarcated with CD11c is markedly accumulated in fat and functions as classically activated M1 macrophages (M1 ATMs) (5, 9). Accumulated M1 ATMs in obese adipose tissue contribute to the increased expression levels of proinflammatory cytokines, such as TNFα and IL-6, in adipose tissue, which is mechanistically linked to insulin resistance (2, 5, 9, 10). It has been shown that blockade of ATM accumulation by inhibition of monocyte trafficking during obesity prevents obesity-induced adipose tissue inflammation and glucose intolerance (11, 12). Furthermore, ablation of CD11c^+^ ATM in obese adipose tissue attenuates adipose tissue inflammation and improves in glucose tolerance, supporting the importance of M1 ATMs in obesity-induced adipose tissue inflammation (13, 14). However, the molecular mechanism that underlies the polarization and maintenance of the proinflammatory M1 ATMs in obesity has not been fully elucidated.

The nuclear lamina is a protein meshwork that surrounds and protects the nuclear content. In addition to providing the structural scaffold of the nucleus, the nuclear lamina is involved in diverse cellular functions, including chromatin organization, DNA replication and repair, transcription, and nuclear migration (15, 16). Lamins, type V intermediate filament, are the major components of the nuclear lamina. So far, seven lamin isoforms have been reported in mammals and are grouped into A-type and B-type lamins based on their biochemical and immunological properties (17, 18). In the rodent model, alternative splicing of the single *Lmna* gene produces all A-type lamins, including lamin A, lamin C, lamin AΔ10, and lamin C3. In most somatic cells, lamin A and C are coexpressed and are the major isoforms among A-type lamins. *Lmnb1* encodes lamin B1, while *Lmnb2* expresses lamin B2 and lamin B3 through alternative splicing. Lamin A and C are rarely expressed in cells at early developmental stages and lack in some somatic cells in adulthood, whereas lamin B1 and B2 are expressed in most cells throughout development (19, 20). *Lmnb1* or *Lmnb2* knockout mice die at birth with defects in multiple tissues (21–23), whereas *Lmna* knockout mice are born apparently normal, but die 16–18 days after birth (24, 25). In humans, mutations in *LMNA* are associated with a range of diseases, including lipodystrophy, cardiomyopathy, muscular dystrophy, and progeria.

Previous studies have shown that alterations of the nuclear lamina are associated with increased immune responses and metabolic disorders in humans (26, 27). In particular, Dunningan-type lipodystrophy characterized by mutations in *LMNA* shares many features of the metabolic syndrome (28). Genome-wide association studies have identified that genetic variants in *LMNA* are linked with type 2 diabetes in several populations (29–32). Moreover, Miranda et al. found that lamin A/C expression is upregulated in adipose tissue in obese and type 2 diabetes patients (33).

Based on the importance of adipose tissue inflammation in obesity-associated metabolic dysfunction and the linkage of lamins with metabolic disorder, we hypothesize that lamins in ATMs play a role in the development of obesity-induced adipose tissue inflammation and, thereby, insulin resistance. Herein, we show that obesity increases lamin A/C expression in adipose tissue in both rodent models and human. In adipose tissue, lamin A/C is specifically upregulated in ATMs, in particular in CD11c^+^ M1 ATMs, by obesity. We further demonstrate that overexpression of lamin A/C in macrophages promotes proinflammatory cytokine gene expression by enhancing NF-κB activity, while depletion of lamin A/C in macrophages suppresses LPS-induced inductions of proinflammatory genes. Moreover, myeloid cell-specific deletion of *Lmna* improves obesity-induced insulin resistance and adipose tissue inflammation. Hence, these data strongly suggest that lamin A/C in ATMs plays an important role in the regulation of obesity-induced inflammation and insulin resistance.

## Materials and Methods

### Animal Studies

C57BL/6J male mice were purchased from Orient Bio in Korea. Mice were *ad libitum* fed with a normal diet (ND, 4.5% fat; PMI Nutrition International) or a high-fat diet (HFD) consisting of 60% fat (Research Diets) beginning 6 weeks of age for a duration of 12 weeks. *Ob/ob* and *db/db* mice were purchased from Central Laboratory Animal Inc., in Korea and were sacrificed at 10 weeks of age. Glucose tolerance tests were performed after 6 h of fasting. Mice were intraperitoneally injected with glucose (0.7 g/kg) and blood glucose levels were measured at the different time points. In *Lmna^flox/flox^* mice, the exon 2 of *Lmna* is cleaved upon Cre expression (25). Myeloid cell-specific *Lmna* knockout mice (hereafter referred to as MKO) were generated by crossing *Lmna^flox/flox^* mice with LysM-Cre mice. Cre-negative *Lmna^flox/flox^* littermates were used as control mice. All mouse procedures were approved by the Institutional Animal Care and Use Committee at the Soonchunhyang University (SCH16-0003, SCH17-0008).

### Human Studies

The clinical studies were reviewed and approved by the Soonchunhyang University Institutional Review Board (SCH#1040875-201502-BR-009) and were carried out in accordance with the Declaration of Helsinki. Informed personal consents were obtained from all subjects. Omental white adipose tissues were obtained from patients who underwent bariatric surgery in Soonchunhyang Hospitals, Korea. The clinical and biochemical parameters are presented in Table S1 in Supplementary Material.

### Flow Cytometry Analysis of Adipose Tissue Stromal Vascular Cells (SVCs)

Stromal vascular fractions (SVFs) and adipocyte fractions from adipose tissues were isolated as previously described (34). In brief, epididymal white adipose tissues (eWAT) were minced and digested in digestion buffer (0.5% BSA, 25 mM HEPES, 50% HBSS, 50% PBS, and 1 mM EDTA) with collagenase (1 mg/ml). Suspensions were incubated at 37°C for 30 min with intermittent shaking. Then, suspensions were centrifuged at 500 × *g* for 10 min at 4°C. After centrifugation, the floating adipocyte fractions were carefully collected in separate tubes. The pellet was resuspended in RBC lysis buffer and neutralized by adding PBS. The suspension was centrifuged at 500 × *g* for 10 min at 4°C. The pellets (SVFs) were used for gene and/or protein expression analysis. For the flow cytometry analysis, cells were resuspended in staining buffer (0.1% BSA in PBS), incubated in Fc Block (Invitrogen) for 10 min on ice, and then stained with antibodies against cell-specific markers for 30 min at 4°C (Table S2 in Supplementary Material). Stained cells were washed in staining buffer and fixed in 0.1% paraformaldehyde (PFA) before analysis. For intracellular staining, cells were permeabilized and stained by using the Intracellular Fixation and Permeabilization Buffer Set (eBioscience). Cells were analyzed on a FACSCanto II Flow Cytometer (BD Biosciences) using FlowJo software (FlowJo). For sorting cells, SVFs were suspended in RPMI 1640/2% FBS and ATMs were isolated by FACSAria III (BD Bioscience).

### Bone Marrow-Derived Macrophages (BMDMs) and Peritoneal Macrophages

Mouse BMDMs were derived from femoral and tibial bone marrow cells of mice. Briefly, after lysis of RBCs, bone marrow cells were differentiated in BMDM medium (RPMI 1640 containing 10% FBS, 20% L929-conditioned medium, 25 mM HEPES, and 2 mM glutamine) for 6 days. The cells were trypsinized and plated for the treatments or transfections. For the preparation of the peritoneal macrophages, mice were intraperitoneally injected with the thioglycolate solution and cells were harvested 4 days after injection by peritoneal lavage. Cells were plated in RPMI media containing 10% FBS and non-adherent cells were removed by washing with PBS. The remaining cells were used as peritoneal macrophages. For LPS treatment, a final concentration of 10 ng/ml LPS was added and incubated for 2 h.

### Ectopic Expression of Lamin A/C in Macrophages

To generate lamin A/C overexpressing construct, mouse *Lmna* cDNA was amplified from IMAGE clone 4240057. The PCR product was cloned into pEGFP-C3 (Clontech) that overexpressed gene of interest fused with the EGFP gene under a CMV promoter. The resulting construct, pEGFP-C3-*Lmna*, was confirmed by DNA sequencing. pEGFP-N1 that expressed EGFP alone was used as a control. Raw 264.7 cells and BMDMs were transfected with pEGFP-C3-*Lmna* or pEGFP-N1 using Lipofectamine LTX with Plus Reagent (Thermo Fisher) and the Amaxa Nucleofector (Lonza), respectively. NF-κB activity was measured by using a Dual Luciferase Assay kit (Promega). For the luciferase assay, HEK293 cells, HeLa cells, or Raw 264.7 cells were transfected with pEGFP-C3-*Lmna* or pEGFP-N1. Ten hours after transfection, cells were re-transfected with plasmids encoding luciferase reporter gene *luc2P* containing κB binding elements in the promoter and pRL-SV40. The following day, cells were treated with 10 ng/ml LPS for 30 min and lysed and NF-κB activity was measured according to manufacturer’s instructions.

### Gene Expression Analysis

RNA from tissues and cells was extracted by using Trizol Reagent (Life Technologies). cDNA was synthesized from 0.5 to 1.0 µg of total RNA using the High-Capacity cDNA Reverse Transcription Kit (Applied Biosystems). PowerUp SYBR Green PCR Master Mix (Applied Biosystems) and the Step One Plus System (Applied Biosystems) were used for quantitative real-time RT-PCR (qRT-PCR). *Arbp* or 18S expression was used as an internal control for data normalization. Samples were assayed in duplicate and relative expression was determined using the 2^−ΔΔCT^ method. PCR primers used in this study are listed in Table S3 in Supplementary Material.

### Immunoblot Analysis

Cells were washed with PBS and lysed with RIPA Buffer (50 mM Tris, pH7.4, 150 mM NaCl, 1 mM EDTA, 1 mM MgCl_2_, 1% NP-40, 1% sodium deoxycholate, 1% SDS, and 1× protease inhibitor). Proteins were separated with 10% SDS-PAGE gels and transferred onto nitrocellulose membranes. Rabbit anti-lamin A/C (Santa Cruz), goat anti-lamin B (Santa Cruz), and mouse anti-actin (Sigma) antibodies were used as primary antibodies. Anti-rabbit IgG-HRP (Life Technologies), anti-goat IgG-HRP (Jackson ImmunoResearch), and anti-mouse IgG-HRP (Life Technologies) were used as secondary antibodies. Proteins were visualized with a chemiluminescence imaging system (GE healthcare).

### ELISA

Cell supernatants and blood were collected after treatment and the concentration of IL-6, MCP-1, TNFα in culture supernatants, and plasma were measured with ELISA kits (Life Tech) according to the manufacturer’s instruction.

### Assessment of NF-κB Nuclear Translocation

Bone marrow-derived macrophages or HeLa cells were transfected with pEGFP-C3-*Lmna* or pEGFP-N1 using the Amaxa Nucleofector (Lonza) or Lipofectamine 2000 (Life Tech), respectively. Thirty-six hours after transfection, cells were fixed with 4% PFA in PBS for 10 min at room temperature, washed twice with 0.4% Triton X-100 in PBS, and stained with rabbit anti-NF-κB antibody (Cell Signaling) and anti-rabbit IgG-Rhodamine Red-X (Jackson Immunoresearch). Nucleus was stained with 1 µg/ml Hoechst 33258 (Sigma). Fluorescence images were taken under the same condition, including the same exposure times and light intensity. For BMDMs, cells with nuclear staining were defined by both presence of a clear nuclear signal and the absence of cytoplasmic signal. For HeLa cells, the nuclear translocation index for NF-κB in a cell image was defined as average pixels in nuclear area divided by average pixels in cytoplasmic area in the same cell. Nuclear and cytoplasmic boundaries were determined by overlaying staining images of NF-κB and DNA.

### Statistical Analyses

The results were expressed as mean ± SEM. Group means were compared using unpaired two-tailed *t*-test and the linear dependence between two variables was assessed by determining Pearson’s correlation coefficient “*r*” values. Prims (GraphPad) was used for statistical analysis. All *P*-values of <0.05 were considered statistically significant.

## Results

### Obesity Increases the Expression Level of Lamin A/C in Epididymal White Adipose Tissue

**Figure 1:**
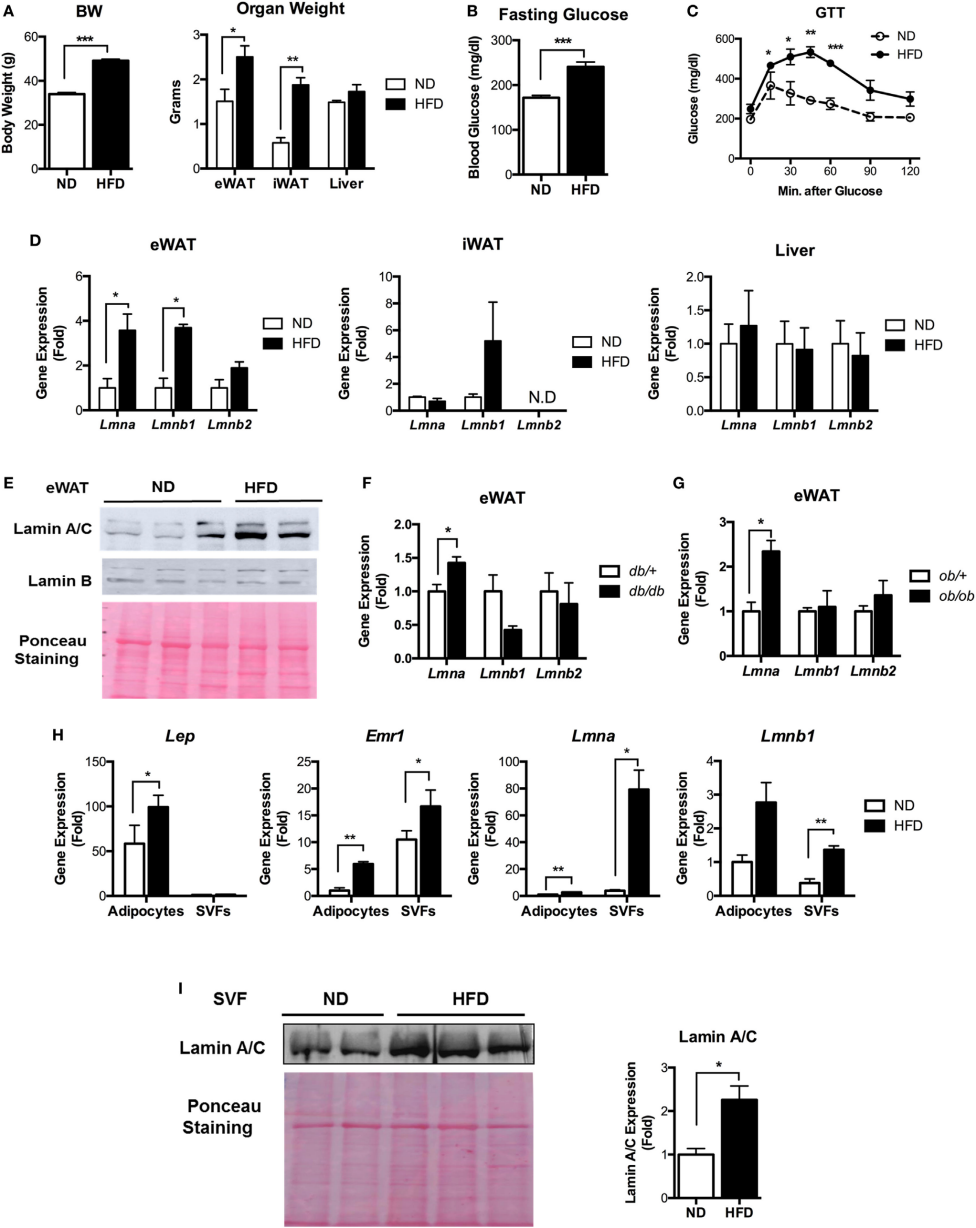
Lamin A/C expression is upregulated in adipose tissue from obese mice. **(A–E)** Male C57BL/6 mice were fed a normal chow diet (ND) or a high-fat diet (HFD) for 12 weeks to induce obesity (*n* = 14 per group). **(A)** Total body weights (left) and organ weights (right), **(B)** fasting blood glucose, **(C)** glucose tolerance test, and **(D)** quantitative real-time RT-PCR (qRT-PCR) analysis of *Lmna, Lmnb1*, and *Lmnb2* in eWAT, iWAT, and liver from ND- and HFD-fed mice. Amounts of transcripts for each gene in HFD tissues relative to those in ND tissues are presented. **(E)** Western blotting analysis of epididymal white adipose tissues (eWAT) lysate from ND- and HFD-fed mice. Lamin A/C (upper), lamin B1 (middle) protein levels are presented. Equal amount of total proteins as measured by Ponceau S staining (lower) of each lane. **(F)** qRT-PCR analysis of *Lmna, Lmnb1*, and *Lmnb2* in eWAT from *db/*+ and *db/db* male mice (8 weeks, *n* = 5 per group). **(G)** qRT-PCR analysis of *Lmna, Lmnb1*, and *Lmnb2* in eWAT from *ob/*+ and *ob/ob* male mice (10 weeks, *n* = 4 per group). **(H)** qRT-PCR analysis of *Lep, Emr1, Lmna*, and *Lmnb1* in adipocyte fractions and stromal vascular cell fractions from eWAT of ND and HFD mice (*n* = 6 per group). **(I)** Immunoblots of lysates from the stromal vascular fraction (SVF) of eWAT from ND and HFD mice for lamin A/C antibody (upper left), Ponceau S staining (lower left) and quantitation of lamin A/C (right) were presented. Error bars represent SEM. *p < 0.05, **p < 0.01, ***p < 0.001.

Since lamin A/C has been linked to type 2 diabetes (26, 32), we examined whether the expression level of lamin A/C is affected by obesity using a diet-induced obesity model. We fed C57BL/6 mice with HFD for 12 weeks. Control mice were fed with normal diet (ND) in parallel with the HFD group. As expected, body weight, adipose tissue weight, and fasting glucose levels were significantly elevated in HFD-fed mice compared to ND-fed mice (Figures 1A,B). HFD-fed mice also appeared to show increased liver weights compared to ND-fed mice. Furthermore, glucose tolerance test (GTT) showed markedly impaired glucose tolerance in HFD-fed mice relative to ND-fed mice (Figure 1C). These results indicate that HFD-fed mice successfully developed obesity and obesity-induced glucose intolerance.

Next, we examined the lamin A/C expression profiles in eWAT, inguinal white adipose tissue (iWAT), and the liver. Quantitative real-time RT-PCR (qRT-PCR) showed that HFD increased the gene expression levels of *Lmna* (gene for lamin A/C) and *Lmnb1* (gene for lamin B1) in eWAT (Figure 1D). The expression level of *Lmnb2* (gene for lamin B2) was not changed in eWAT of the HFD-fed mice (Figure 1D). There were no significant differences in the expression levels of all three lamin genes in iWAT or liver upon HFD treatments (Figure 1D). The expression level of *Lmna* was highest in eWAT among these tissues (Figure S1 in Supplementary Material). Immunoblot analysis confirmed the elevated protein expression of lamin A/C in eWAT of HFD-fed mice compared to ND mice (Figure 1E).

We then examined whether the gene expression levels of lamins were also affected in adipose tissue from genetically obese models, namely, *db/db* and *ob/ob* mice. Consistent with the diet-induced obesity model, the expression level of *Lmna* was significantly increased in both obese *db/db* and *ob/ob* mice compared to their lean controls (Figures 1F,G). There were no notable differences in the gene expression levels of *Lmnb1* and *Lmnb2*. These data show that obesity increases lamin A/C level specifically in the eWAT.

To delineate specific cellular compartments of eWAT with elevated lamin A/C level, we separated adipocyte and SVFs from eWAT of ND- and HFD-fed mice. The expression of the adipocyte-specific marker *Lep* was only found in the adipocyte fraction, while macrophage-specific *Emr1* expression was found in both adipocyte and SVF fractions (Figure 1H). The expression of *Emr1* in the adipocyte fraction was mainly due to contamination of adipose tissue immune cells, in particular ATMs that were strongly bound to the adipocytes and could not be dissociated from adipocytes during the SVF preparation (34–36). qRT-PCR revealed that HFD treatments dramatically increased the gene expression level of *Lmna* in SVFs of eWAT, whereas adipocytes showed a mild increase in *Lmna* expression by the HFD treatment (Figure 1H). This could be mainly due to the contamination of ATMs in the adipocyte fraction. HFD treatments also significantly increased the expression level of *Lmnb1* in SVFs, albeit to a lesser degree than *Lmna*, and showed marginal effects on adipocyte fraction (Figure 1H). Lamin A/C protein level was also markedly increased in SVCs of eWAT from HFD mice compared to that from ND mice (Figure 1I). These results indicate that adipose tissue SVCs are the major cell types that contribute to the elevated levels of lamin A/C in eWAT from obese mice.

### Obesity Upregulates Lamin A/C in M1 ATMs of eWAT

**Figure 2:**
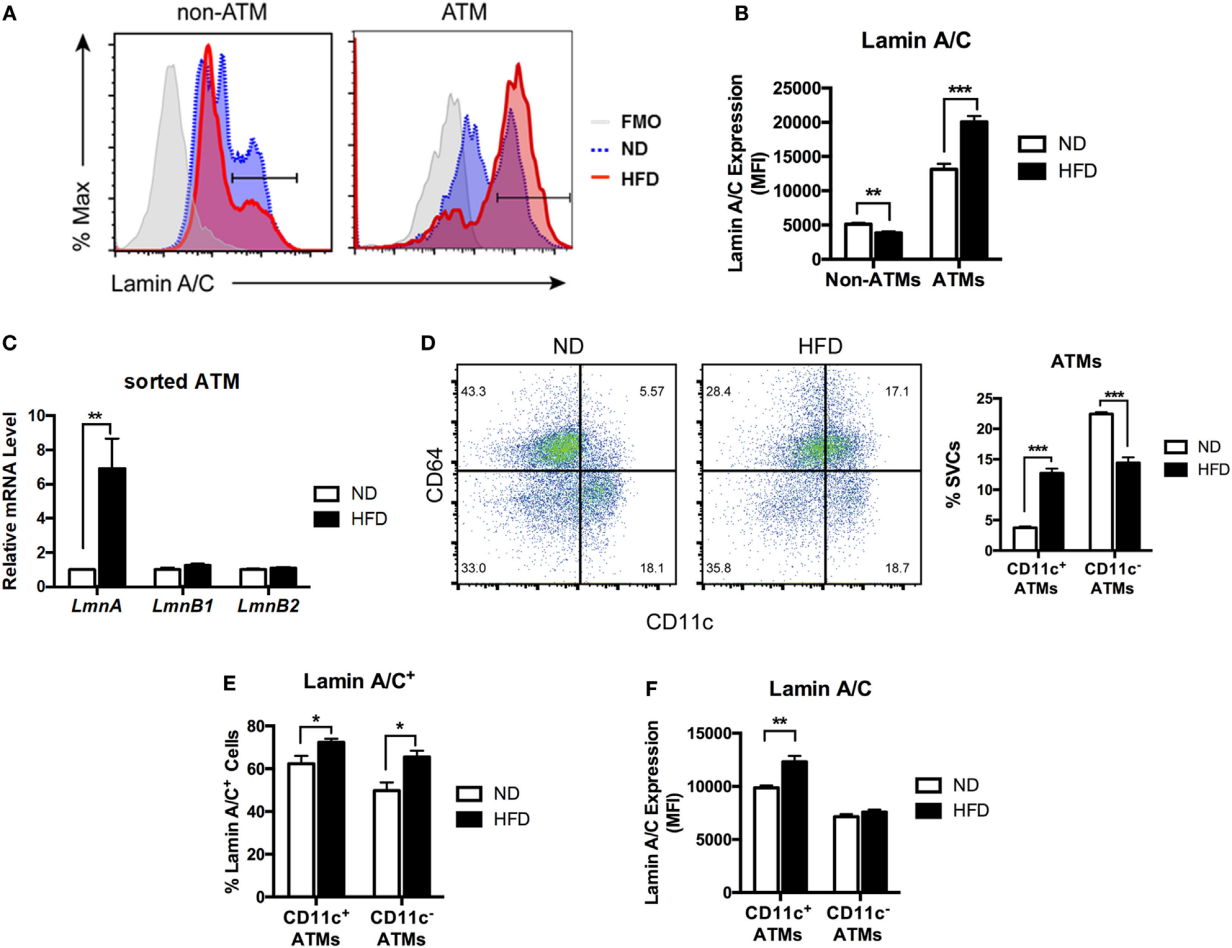
Lamin A/C expression is elevated in adipose tissue macrophages (ATMs) from obese mice. Male C57BL/6 mice were fed a normal diet (ND) or a high-fat diet (HFD) for 12 weeks (*n* = 6 per group). **(A)** Histogram of lamin A/C in non-ATM (CD45^+^CD64^−^) and ATM (CD45^+^CD64^+^) from ND- and HFD-fed mice. **(B)** Lamin A/C expression level as determined by mean fluorescence intensity (MFI) in the non-ATM and ATM populations from epididymal white adipose tissues (eWAT) of ND and HFD mice (*n* = 5 per group). **(C)** Quantitative real-time RT-PCR analysis of *Lmna, Lmnb1*, and *Lmnb2* in sorted ATMs from eWAT of ND- and HFD-fed mice (*n* = 3 per groups). **(D)** A representative flow cytometry plot showing ATMs (CD64^+^CD11c^−^ and CD64^+^CD11c^+^) and ATDCs (CD64^−^CD11c^+^) in stromal vascular cell (left) and frequency of CD11c^+^ ATMs and CD11c^−^ ATMs from eWAT of ND- and HFD-fed mice (right). **(E)** The frequency of lamin A/C^+^ cells in the CD11c^+^ and CD11c^−^ ATM populations in eWAT from ND and HFD mice. **(F)** Lamin A/C expression level as determined by MFI in the CD11c^+^ and CD11c^−^ ATM populations from eWAT of ND and HFD mice. Error bars represent SEM. *p < 0.05, **p < 0.01, ***p < 0.001.

Stromal vascular fraction from adipose tissue contains a variety of cell types, including ATMs, preadipocytes, dendritic cells (DCs), stem cells, T cells, and B cells (14, 37–39). To identify cellular populations responsible for obesity-induced lamin A/C upregulation in SVFs, intracellular lamin A/C staining and flow cytometry analyses were performed on SVFs from eWAT of ND- and HFD-fed mice. In lean state, only about 16% of non-ATM adipose leukocytes expressed lamin A/C, while 55% of ATMs expressed lamin A/C (Figure 2A). HFD treatments further decreased the frequency of lamin A/C expression in non-ATM leukocytes, whereas HFD treatment significantly increased lamin A/C expressing ATMs by about 22% (Figure 2A). Total protein expression levels of lamin A/C, as determined by mean fluorescence intensity (MFI), showed the similar profiles; non-ATM leukocytes expressed much lesser levels of lamin A/C than ATMs in ND mice, and HFD treatments increased lamin A/C expression in ATMs by 50% (Figure 2B). This was further confirmed by qRT-PCR of sorted ATMs. The expression level of *Lmna* was significantly increased over sixfold in sorted ATMs from HFD-fed mice compared to ND-fed mice (Figure 2C). However, the expression levels of *Lmnb1* and *Lmnb2* were not changed in ATMs from HFD-fed mice. These data together suggest that obesity-induced upregulation of lamin A/C in total adipose tissue is mainly contributed by ATMs.

Obesity changes polarization of ATMs by increasing CD11c^+^ M1 ATMs. Hence, we next examined changes in the expression level of lamin A/C in the ATM subpopulations in obesity. Flow cytometry analysis revealed that mice fed a HFD exhibited increased the frequencies of CD11c^+^ M1 ATMs and a decrease in the frequency of CD11c^−^ ATMs compared to those fed with ND (Figure 2D). Furthermore, HFD treatment increased lamin A/C^+^ ATMs in both CD11c^+^ and CD11c^−^ ATM populations compared to the ND-fed mice (Figure 2E). However, HFD treatments significantly increased the protein expression levels of lamin A/C only in CD11c^+^ ATMs, but not in CD11c^−^ ATMs (Figure 2F). These results indicate that lamin A/C is specifically upregulated in ATMs, in particular CD11c^+^ M1 ATMs, by obesity.

### Overexpression of Lamin A/C Induces Inflammation in Macrophages

**Figure 3:**
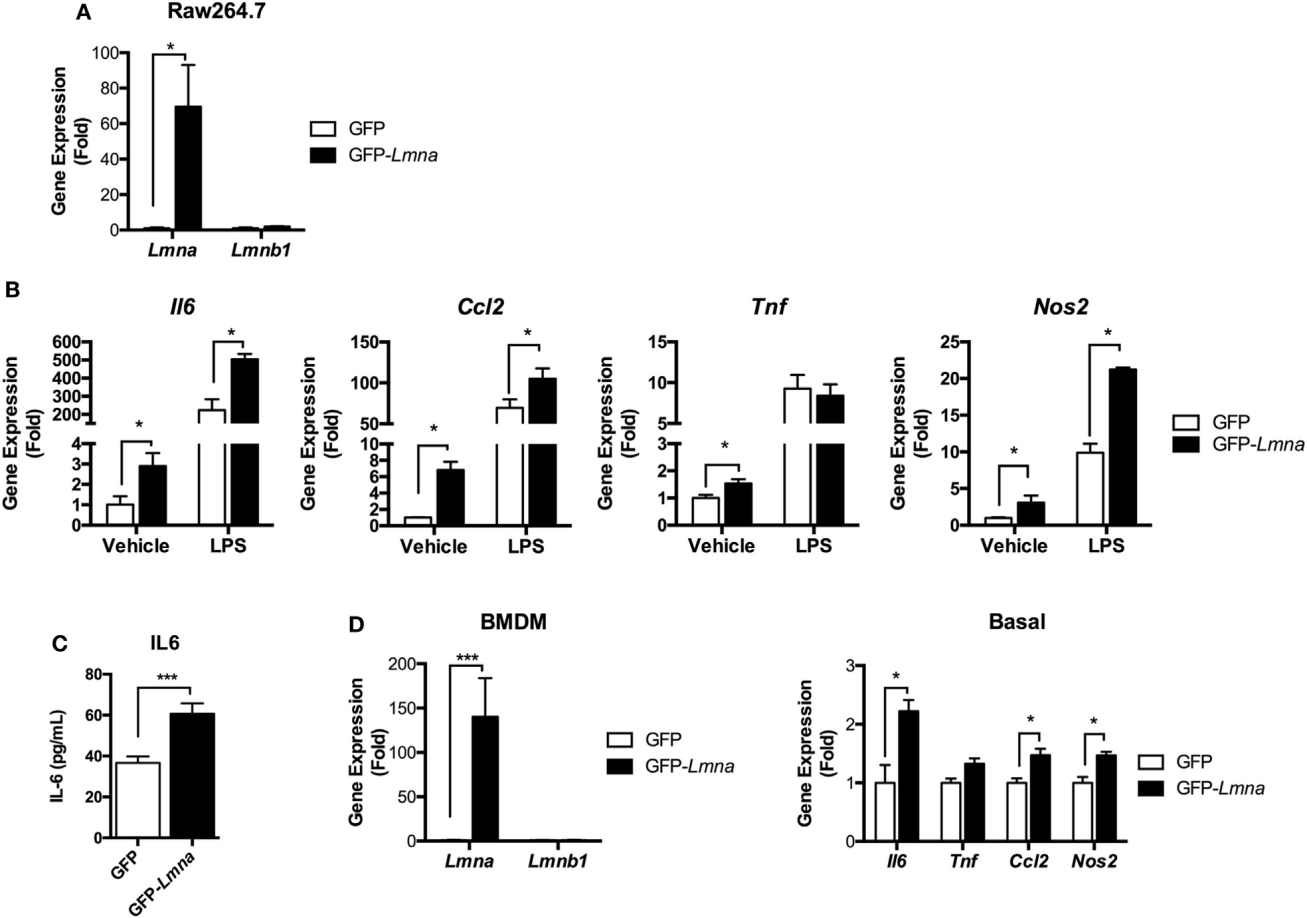
Lamin A/C overexpression induces proinflammatory cytokine expression in macrophages. **(A–C)** Raw 264.7 cells were transfected with GFP or GFP-*Lmna*. After 24 h, transfected cells were cultured in the absence or presence of 100 ng/ml LPS for 6 h. **(A)** Quantitative real-time RT-PCR (qRT-PCR) analysis of *Lmna* and *Lmnb1* in GFP or GFP-*Lmna*-transfected cells. **(B)** qRT-PCR analysis of *Il6, Tnf, Ccl2*, and *Nos2* in GFP or GFP-*Lmna* transfected cells. **(C)** ELISA of IL-6 in supernatant of Raw 264.7 cells transfected with GFP or GFP-*Lmna* in the absence of LPS. **(D)** Characterization of bone marrow-derived macrophages (BMDMs) transfected GFP-*Lmna*. qRT-PCR analysis of *Lmna* and *Lmnb1* (left) and *Il6, Tnf, Ccl2*, and *Nos2* in GFP or GFP-*Lmna* transfected BMDMs (right). Error bars represent SEM. *p < 0.05, ***p < 0.001.

Lamin A/C expression was increased in M1 ATMs by obesity, suggesting that lamin A/C could also play a role in the regulation of inflammation in macrophages. Thus, we first examined this by investigating whether ectopic overexpression of lamin A/C in Raw 264.7 macrophages regulates inflammatory responses. Transfection of GFP-*Lmna* into Raw 264.7 cells overexpressed *Lmna* over the 60-fold. However, the expression level of *Lmnb1* was not affected by transfection of GFP-*Lmna* (Figure 3A). Interestingly, we found that overexpression of lamin A/C spontaneously increased the expression levels of proinflammatory genes, such as *Il6, Tnf, Ccl2*, and *Nos2* even without any stimulation (Figure 3B). LPS treatment further increased expression levels of *Il6, Ccl2*, and *Nos2* in *Lmna*-overexpressing macrophages compared to control cells (Figure 3B). ELISA analysis confirmed that lamin A/C overexpression increased IL6 secretion in the medium without any stimulation (Figure 3C). BMDMs also showed a similar pattern to Raw264.7 cells; transfection of GFP-*Lmna* into BMDMs overexpressed *Lmna* over the 140-fold, but not affecting that of *Lmnb1* (Figure 3D). Moreover, overexpression of lamin A/C significantly increased the expression levels of *Il6, Ccl2*, and *Nos2* (Figure 3D). Collectively, these results indicate that overexpression of lamin A/C increased the expression of proinflammatory genes in macrophages under both basal- and LPS-stimulated inflammatory states.

### Lamin A/C Increases NF-κB Activity *via* Nuclear Translocation of Rel A

**Figure 4:**
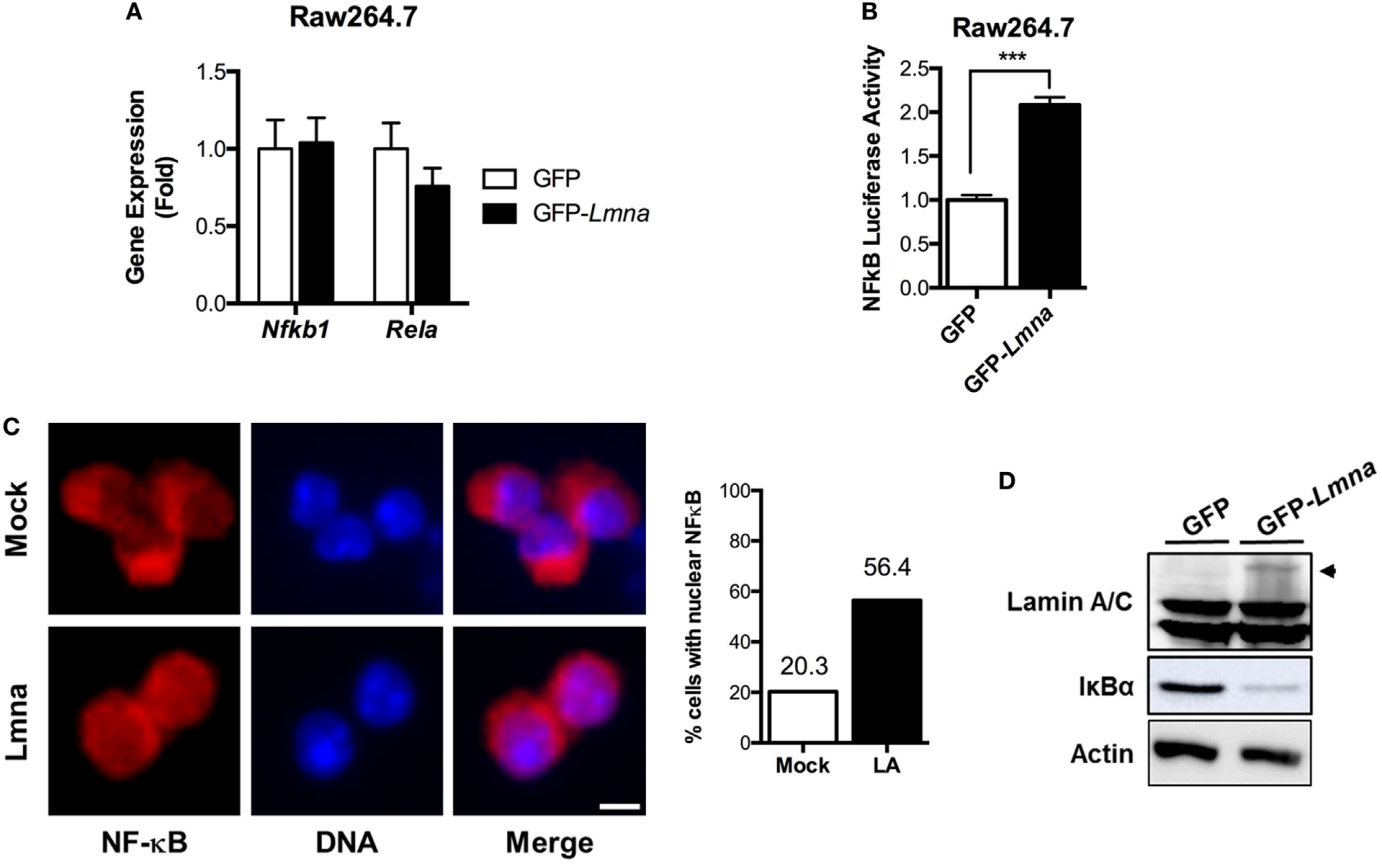
Lamin A/C increases NF-κB activity *via* nuclear translocation of p65/Rel A, a NF-κB complex subunit. **(A)** qRT-PCR analysis of *Nfkb1* and *Rela* in Raw 264.7 cells transfected with either GFP or GFP-*Lmna*. **(B)** NF-κB luciferase activity in Raw 264.7 cells co-transfected with GFP or GFP-*Lmna* together with pNF-κB-Luc, a luciferase reporter construct that has κB responsible elements in the promoter. Luciferase activities were normalized by those in cells co-transfected with a non-reporter plasmid, pRL-SV40. **(C)** Immunofluorescence images of bone marrow-derived macrophages transfected with GFP (Mock) or GFP-*Lmna* (Lmna) and quantitation for cells with nuclear NF-κB p65/Rel A. Cells with nuclear NF-κB were defined based on the presence of nuclear signal and obvious lack of cytoplasmic signal as shown in the representative images (Scale bar: 5 μm). **(D)** Immunoblots of lysates from transfected Raw 264.7 cells with anti-Lamin A/C (top), anti-IκBα (middle), or anti-actin (bottom). Arrowhead marks GFP fusion lamin A protein size.

NF-κB is a master nuclear transcription factor for the genes involved in inflammatory responses. Since our data strongly suggest that lamin A/C regulates gene expression of proinflammatory responses in macrophages, we tested whether lamin A/C also mediates NF-κB functions. We first examined whether ectopic overexpression of *Lmna* changed the mRNA levels of NF-κB p105 and p65/Rel A subunits. qRT-PCR analysis revealed that overexpression of lamin A/C did not affect the gene expression levels of NF-κB p105 and p65/Rel A subunits (Figure 4A). We then measured NF-κB transcriptional activity by using a luciferase system. We found that transfecting *GFP-Lmna* into Raw 264.7 cells significantly increased NF-κB transcriptional activity even without any stimulation by LPS (Figure 4B). Increase of basal NF-κB transcriptional activity was also shown in lamin A/C overexpressing HEK293 cells and HeLa cells (Figures S2A,B in Supplementary Material). In HeLa cells, LPS treatment modestly increased NF-κB activity in control cells and lamin A/C overexpression further increased NF-κB activity (Figure S2B in Supplementary Material).

We also examined nuclear translocation of endogenous Rel A in BMDM by using immunofluorescence with anti-Rel A antibody, which is another established method to determine NF-κB activation. Immunofluorescence analysis revealed that, in mock control BMDMs, Rel A signal was preferentially localized in the cytoplasm, whereas Rel A was enriched in the nucleus of BMDMs transfected with GFP-*Lmna* (Figure 4C). Quantitative counting of cells with nuclear Rel A staining revealed that overexpression of lamin A/C in BMDMs increased the nuclear translocation of Rel A by ~2.8-folds compared to the mock controls (Figure 4C). Similarly, the increase of nuclear translocation of Rel A was also observed in HeLa cells that were transfected with GFP-*Lmna* compared to control cells in both basal and TNFα-stimulated condition (Figures S2C,D in Supplementary Material). Nuclear translocation and thus activation of NF-κB are tightly regulated by the IKKβ/IκBα/NF-κB pathway. Under the basal condition, IκBα binds to NF-κB and, therefore, sequesters NF-κB in cytoplasm. However, when IKKβ is activated by stimulants, such as LPS or TNFα, IKKβ phosphorylates IκBα. Phosphorylated IκBα is poly-ubiquitinated and degraded in a proteasome-dependent way. This releases NF-κB from IκBα sequestration, and free NF-κB can be translocated into nucleus. Thus, measurements of IκBα protein amounts can be used as a surrogate marker for the activation of IKKβ. We found that overexpression of lamin A/C in BMDMs markedly decreases IκBα protein amount (Figure 4D), indicating that overexpression of lamin A/C activates the IKKβ/IκBα/NF-κB pathway. These data together show that lamin A/C overexpression induces NF-κB activation by activating the IKKβ/IκBα/NF-κB pathway in both basal and stimulatory conditions.

### Depletion of Lamin A/C Suppresses LPS-Induced Inflammation in Macrophages

**Figure 5:**
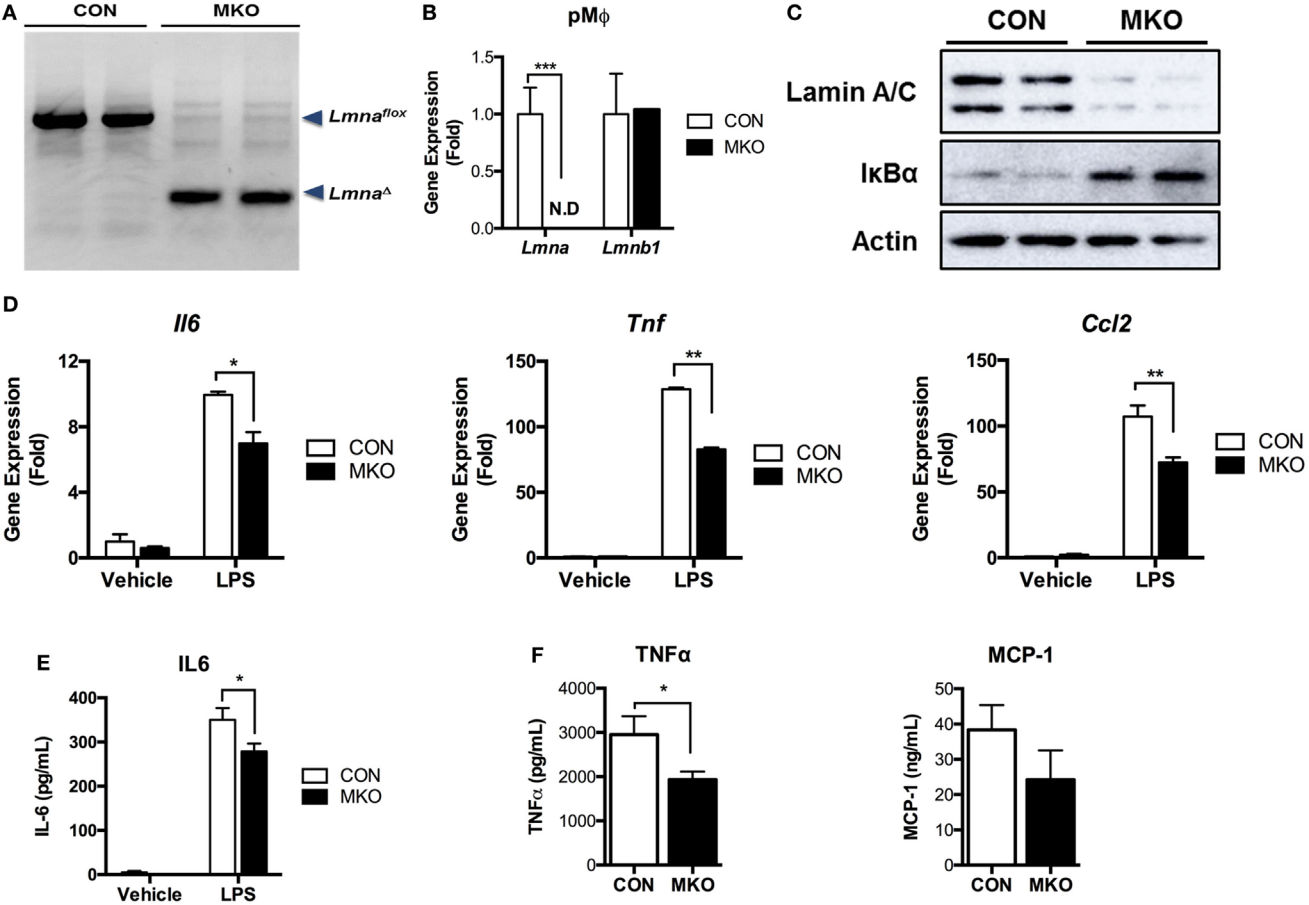
Depletion of lamin A/C suppresses proinflammatory gene activation upon LPS treatment in macrophages. **(A–E)** Analyses of peritoneal macrophages from control and myeloid cell-specific *Lmna* KO mice (MKO) mice. **(A)** PCR genotyping of peritoneal macrophages from WT control (CON, *Lmna^flox/flox^*) and MKO (LysM-Cre; *Lmna^flox/flox^*) mice. Arrowheads mark *Lmna^flox^* (uncleaved) and *Lmna*^Δ^ (cleaved) alleles. **(B)** qRT-PCR analysis of *Lmna* and *Lmnb1* in peritoneal macrophages isolated from CON and MKO mice. **(C)** Immunoblots of lysates from peritoneal macrophages for lamin A/C (top), IκBα (middle), or actin (bottom). **(D,E)** Peritoneal macrophages were isolated from control and MKO mice and then treated with vehicle or 10 ng/ml LPS for 2 h. **(D)** qRT-PCR analysis of *Il6, Tnf*, and *Ccl2* in peritoneal macrophages isolated from CON and MKO mice. **(E)** Level of IL-6 in supernatant of CON and MKO peritoneal macrophages treated with vehicle or 10 ng/ml LPS for 6 h. **(F)** Plasma TNFα and MCP-1 levels after i.p. injection of LPS (20 mg/kg BW) were measured in CON and MKO mice (*n* = 6 per group). Error bars represent SEM. *p < 0.05, **p < 0.01.

Next, we examined whether deleting *Lmna* conversely suppressed inflammatory responses in macrophages. We isolated peritoneal macrophages from control WT (CON) mice and myeloid cell-specific *Lmna* KO mice (MKO). PCR genotyping of peritoneal macrophages confirmed that *Lmna^flox^* alleles were cleaved in macrophages isolated from MKO mice (Figure 5A). *Lmna^flox^* alleles remained uncleaved in all other tested tissues from MKO mice, suggesting that there was no or minimal leakage of LysM-Cre expression (data not shown). MKO mice showed almost complete depletion of *Lmna* mRNA in peritoneal macrophages without showing any compensatory increases in *Lmnb1* expression (Figure 5B). We then measured the IκBα protein amount to assess the activation of the IKKβ/IκBα/NF-κB pathway. We found that deletion of *Lmna* in macrophages significantly increased IκBα protein amount (Figure 5C), suggesting that depletion of lamin A/C inhibits IKKβ activity and, therefore, suppresses NF-κB activation. To test the role of lamin A/C in the regulation of proinflammatory gene expressions, we isolated peritoneal macrophages from CON and MKO mice and treated with LPS. We found that the basal expression levels of *Il6, Tnf*, and *Ccl2* were similar in both genotypes (*p* > 0.05) (Figure 5D). However, LPS-induced expressions of these genes were significantly lower in MKO peritoneal macrophages than those in the peritoneal macrophages from CON mice (Figure 5D). Secretion of IL-6 after LPS treatment was also lower in peritoneal macrophages from MKO mice than that of CON mice (Figure 5E). To test whether deleting *Lmna* conversely suppressed inflammatory responses *in vivo*, LPS were injected to CON and MKO mice. After 6 h injection, bloods were collected and levels of inflammatory cytokines in plasma were measured. Compared to CON, MKO lowered circulating TNFα and MCP-1 levels (Figure 5F). Overall, these data together demonstrate that lamin A/C regulates proinflammatory responses in macrophages.

### Myeloid Cell-Specific Lamin A/C Deficiency Improves Obesity-Induced Insulin Resistance and Adipose Tissue Inflammation

**Figure 6:**
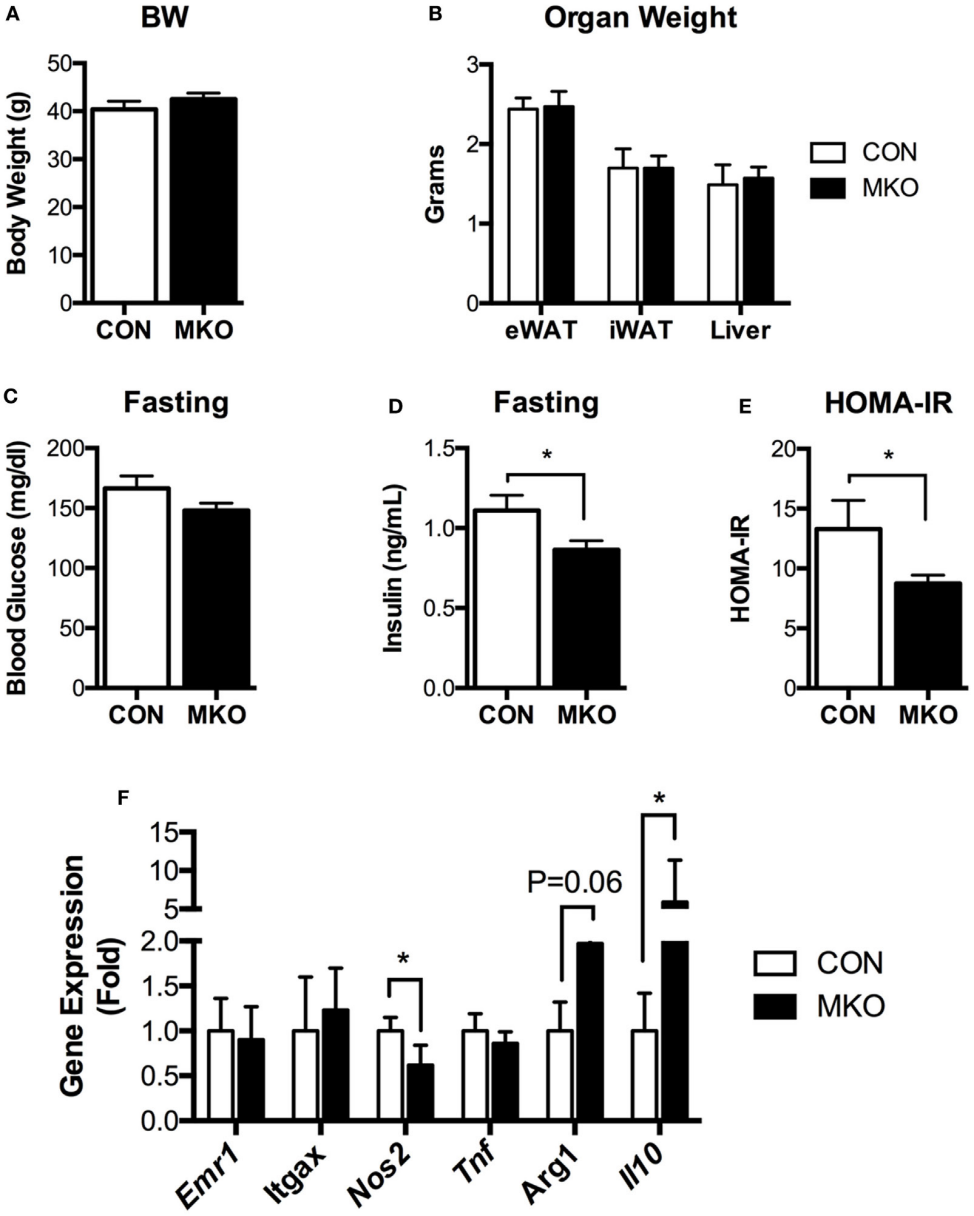
Deletion of *Lmna* in myeloid tissues attenuates high-fat diet-induced insulin resistance in mice. CON and myeloid cell-specific *Lmna* KO mice (MKO) male mice (6 weeks) were fed a high-fat diet (HFD) for 12 weeks (CON, *n* = 4; MKO, *n* = 9). **(A)** Body weight, **(B)** organ weights of epididymal white adipose tissues (eWAT), inguinal white adipose tissue, and liver, **(C)** fasting blood glucose, **(D)** fasting plasma insulin, **(E)** homeostatic model assessment for insulin resistance index, **(F)** gene expression in eWAT from CON and MKO mice. Error bars represent SEM. *p < 0.05.

Given that lamin A/C was elevated in obese adipose tissue and lamin A/C regulated inflammation in macrophages, we then examined the role of myeloid cell lamin A/C in the development of obesity-induced inflammation and insulin resistance. Control and MKO mice were fed with a HFD for 12 weeks. MKO mice showed similar body weight and adipose tissue weight compared to control mice (Figures 6A,B). However, HFD-fed MKO mice showed lower fasting glucose and insulin levels (Figures 6C,D) and, therefore, improved insulin resistance as determined by homeostatic model assessment for insulin resistance (HOMA-IR) (Figure 6E), indicating that deletion of *Lmna* in macrophages improves obesity-induced systemic insulin resistance. Gene expression analysis of total eWAT showed that deletion of *Lmna* in macrophages increased the expression levels of anti-inflammatory genes, including *Arg1* and *Il10*, and decreased the expression level of the proinflammatory gene *Nos2* (Figure 6F). However, *Lmna* deletion in macrophages did not affect the gene expression levels of *Emr1* and *Itgax*, which are macrophage-specific markers, or *Tnf* in the total adipose tissue.

### The Expression Level of *LMNA* Correlates With BMI and *IL6* Gene Expression in Human Adipose Tissue

**Figure 7:**
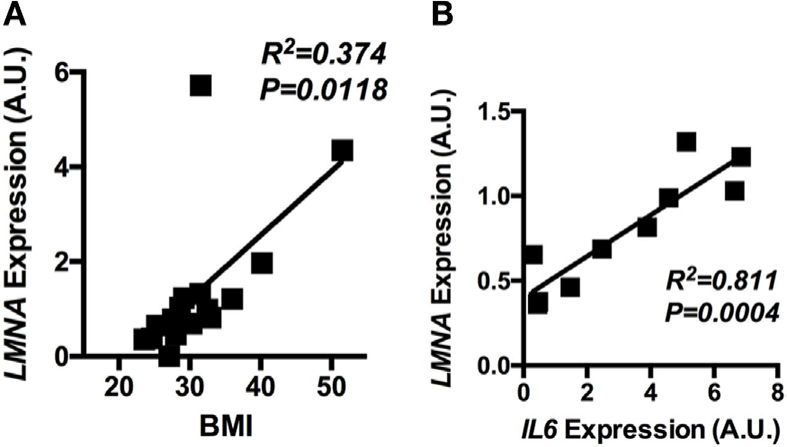
The adipose *LMNA* expression levels correlate with BMI and *IL6* expressions in human subjects. Human omental adipose tissue samples were obtained by surgical biopsy from 30 individuals, and *LMNA* and *IL6* mRNA levels were determined by quantitative real-time RT-PCR. Linear regression analyses of *LMNA* mRNA level in human omental adipose tissue with respect to **(A)** BMI and **(B)** *IL6* mRNA levels are presented. Pearson’s correlation coefficients (*r*) between *LMNA* and either BMI or *IL6* level are presented in each graph.

Having shown that lamin A/C was upregulated in obese WAT and had an inflammatory role in mouse macrophages, mRNA expression of lamin A/C in human visceral adipose tissue was further examined in relation to BMI. qRT-PCR analysis showed that the expression level of *LMNA* was significantly elevated in obese individuals as compared with lean or overweight subjects (data not shown). Correlation data showed that *LMNA* expression in visceral fat is positively associated with BMI (*p* = 0.0118, Figure 7A). We also found that *LMNA* expression was positively correlated with *IL6* expression in the same set of human adipose tissue (*p* = 0.0004, Figure 7B).

## Discussion

Adipose tissue macrophages play an important role in the regulation of adipose tissue inflammation in obesity and the development of metabolic syndromes. There has been considerable interest in identifying regulatory mechanisms to activate ATMs and maintain their proinflammatory function of ATMs in obesity. Since lamins are associated with metabolic syndromes, the goal of this study is to investigate the role of lamins in adipose tissue inflammation and systemic insulin resistance. In this study, we show that among the lamin isoforms, lamin A/C is specifically upregulated in visceral WAT of obese humans and mouse models. Importantly, lamin A/C is enhanced particularly in obese ATMs in mouse models. Furthermore, lamin A/C overexpression in macrophages leads to the upregulation of proinflammatory genes, such as *Tnf, Il6, Nos2*, and *Ccl2* by activating the IKKβ/IκBα/NF-κB pathway. Moreover, specific deletion of *Lmna* in myeloid cells not only suppresses proinflammatory responses in macrophages, but also improves obesity-induced systemic insulin resistance. These observations suggest that lamin A/C in ATMs functions as a novel regulator in obesity-induced adipose tissue inflammation and insulin resistance.

There are several studies showing the increased lamin A/C expression in adipose tissue in obesity (33, 40, 41). Miranda et al. (33) reported increased lamin A/C mRNA level in adipose tissues from obese human subjects. Independent research group also found that lamin A/C levels were elevated in adipose tissues from both obese individuals and *ob/ob* mice (41). However, these studies did not identify the cellular compartment of adipose tissue in which lamin A/C expression is elevated. In agreement with previous studies, we found that visceral adipose tissue from obese subjects and eWAT from the various obese mouse models have higher expression levels of lamin A/C. Closer examination reveals that the expression levels of lamin A/C is increased mainly in ATMs. Interestingly, induction of lamin A/C is only shown in eWAT, but not in iWAT. It has been known that visceral fat of human or eWAT of rodent models contains more inflammatory cells compared to subcutaneous fat in obese humans and rodents (42–44). Furthermore, the expression levels of proinflammatory genes are higher in eWAT than in iWAT (2). These observations suggest that the upregulation of lamin A/C in eWAT is more tightly associated with inflammation than obesity *per se*. This could explain the association of *LMNA* with *IL6* expression in human adipose tissue observed in this study.

Our results from *Lmna* gain- and loss-of-function experiments in macrophages reveal that lamin A/C is a novel regulator in the production of proinflammatory mediators, such as TNFα, IL-6, and CCL2, in line with the role of lamin A/C in immune response. In obesity, ATMs accumulate and are activated to produce high level of proinflammatory cytokines, which lead to adipose tissue inflammation and insulin resistance (5, 45). Several studies showed that inhibitions of macrophage activation and proinflammatory cytokine functions improve insulin resistance (46–48). Our results show that lamin A/C expression was upregulated in ATMs and played a role in the production of proinflammatory genes, which immediately raises a question whether lamin A/C upregulation in ATMs contributes to the development of the obesity-induced inflammation and insulin resistance. Indeed, our data with obese MKO mice showed the improvement of insulin resistance upon HFD. The notion of the immunological role of lamin A/C in chronic inflammation is consistent with recent reports showing that age-associated systemic inflammation is linked to the fluctuation of the nuclear lamina (49, 50).

NF-κB pathway has been known as a master regulator in the immune response. In this study, we homed in on the effect of lamin A/C overexpression on NF-κB activity. Since there was no change in the mRNA levels of NF-κB p105 and p65/Rel A at the basal state (Figure 4A) and lamin A did not directly interact with RelA (data not shown), we surmised that lamin A/C overexpression leads to increased Rel A nuclear translocation. Our studies show that NF-κB transcriptional activity and Rel A nuclear translocation were enhanced in lamin A/C overexpressing macrophages, which was in parallel with higher levels of *Il6* and *Ccl2* in these cells. Importantly, even without any stimulation, nuclear localization of NF-κB was increased (Figures S2C,D in Supplementary Material), and this translated into the induction of NF-κB transcriptional activity and proinflammatory gene expression without any other stimulations (Figure 3; Figures S2A,B in Supplementary Material). The question is how lamin A/C can induce NF-κB translocation. However, we found evidence in identifying a potential molecular mechanism for this. Since IKKβ phosphorylates IκBα and this induces degradation of IκBα, we used the assessment of IκBα protein amounts as a surrogate marker for IKKβ activation. We found that overexpression of lamin A/C decreased IκBα protein amount (Figure 4D) and that deletion of lamin A/C conversely increased IκBα protein amount (Figure 5C), suggesting that lamin A/C regulates IKKβ activity. Thus one of the potential scenario how lamin A/C controls inflammation is that (1) lamin A/C activates IKKβ, and induces NF-κB nuclear translocation and activation in the basal state, (2) this, in turn, increases the expression levels of proinflammatory genes, including *Tnf*, (3) these proinflammatory cytokines then activate the IKK/IκBα/NF-κB pathway, and (4) thus this initiates the continuous vicious cycle of activation for this pathway and, therefore, induces inflammation. Supporting this is the previous study showing that accumulation of prelamin A at the nuclear lamina activates the NF-κB pathway by promoting NEMO-dependent signaling or inflammasome formation (49, 51). Another potential mechanism is the regulation of nuclear NF-κB transcriptional activity by reorganizing chromatin structure. Recent studies have been shown that the 3D genome organization undergoes dramatic changes during immune activation. A genome-wide study shows that release of immune gene loci from the nuclear periphery is important for the activation of immune responses (52). Overproduction of lamin A/C may also lead to alteration of 3D chromatin organization and/or genome interaction with the nuclear periphery causing deregulated expression of inflammatory genes. We could not also exclude the possibility for the regulation of actin dynamics by lamin A/C, since this has been identified as one mechanism by which lamin regulates the nuclear translocation and downstream signaling of transcription factor megakaryoblastic leukemia 1 (53). It is also possible that lamin A/C overexpression simply increases retention of Rel A in the nucleus in the context of obesity, which could lead to NF-κB hyperactivation, and thereby further aggravating the inflammatory cascade in already inflamed adipose tissue. Further study is needed to investigate the detailed mechanisms of lamin A/C-induced NF-κB activation and its pathophysiological relevance *in vivo*.

In summary, we show that the expression of lamin A/C, a component of nuclear lamina, is specifically upregulated in obese adipose tissue, which is largely attributed to ATMs. We also show that lamin A/C regulates proinflammatory responses *via* NF-κB activity and myeloid-specific lamin A/C deletion improves obesity-induced inflammation and insulin resistance. Thus, our data suggest that lamin A/C mediates the activation of ATM inflammation by regulating NF-κB, thereby contributing to the development of obesity-induced insulin resistance.

## Ethics Statement

All mouse procedures were approved by the Institutional Animal Care and Use Committee at the Soonchunhyang University (SCH16-0003, SCH17-0008). The clinical studies were reviewed and approved by the Soonchunhyang University Institutional Review Board (SCH#1040875-201502-BR-009), and carried out in accordance with the Declaration of Helsinki. Informed personal consents were obtained from all subjects.

## Author Contributions

YK, JL, and KC conceived the idea. PB, JC, and YP performed animal experiments and cell culture experiments. MK and AB performed Rel A nuclear translocation and NF-κB luciferase assay experiments. SH, Y-JK, CC, and WK contributed to analysis of human studies. YK, JL, and KC interpreted the data and wrote the manuscript.

## Conflict of Interest Statement

The authors declare that the research was conducted in the absence of any commercial or financial relationships that could be construed as a potential conflict of interest.
