# Supplementary figures and images for: Macrophage Lamin A/C Regulates Inflammation and the Development of Obesity-Induced Insulin Resistance
Source: Front Immunol. 2018 Apr 20;9:696. doi: 10.3389/fimmu.2018.00696 (PMC5920030; doi:10.3389/fimmu.2018.00696)

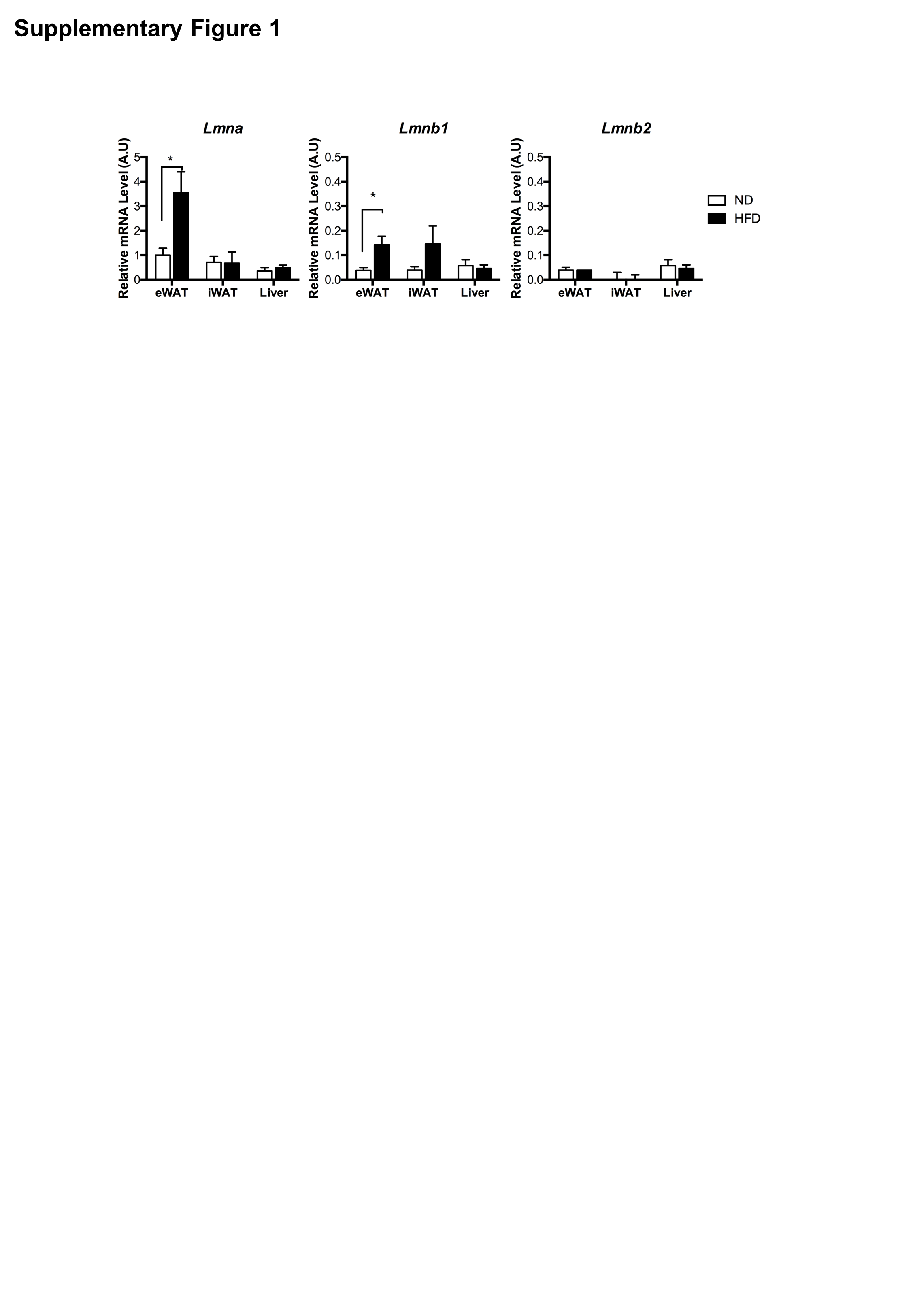

Supplement: Supplementary file 1 [file Image_1.TIF]

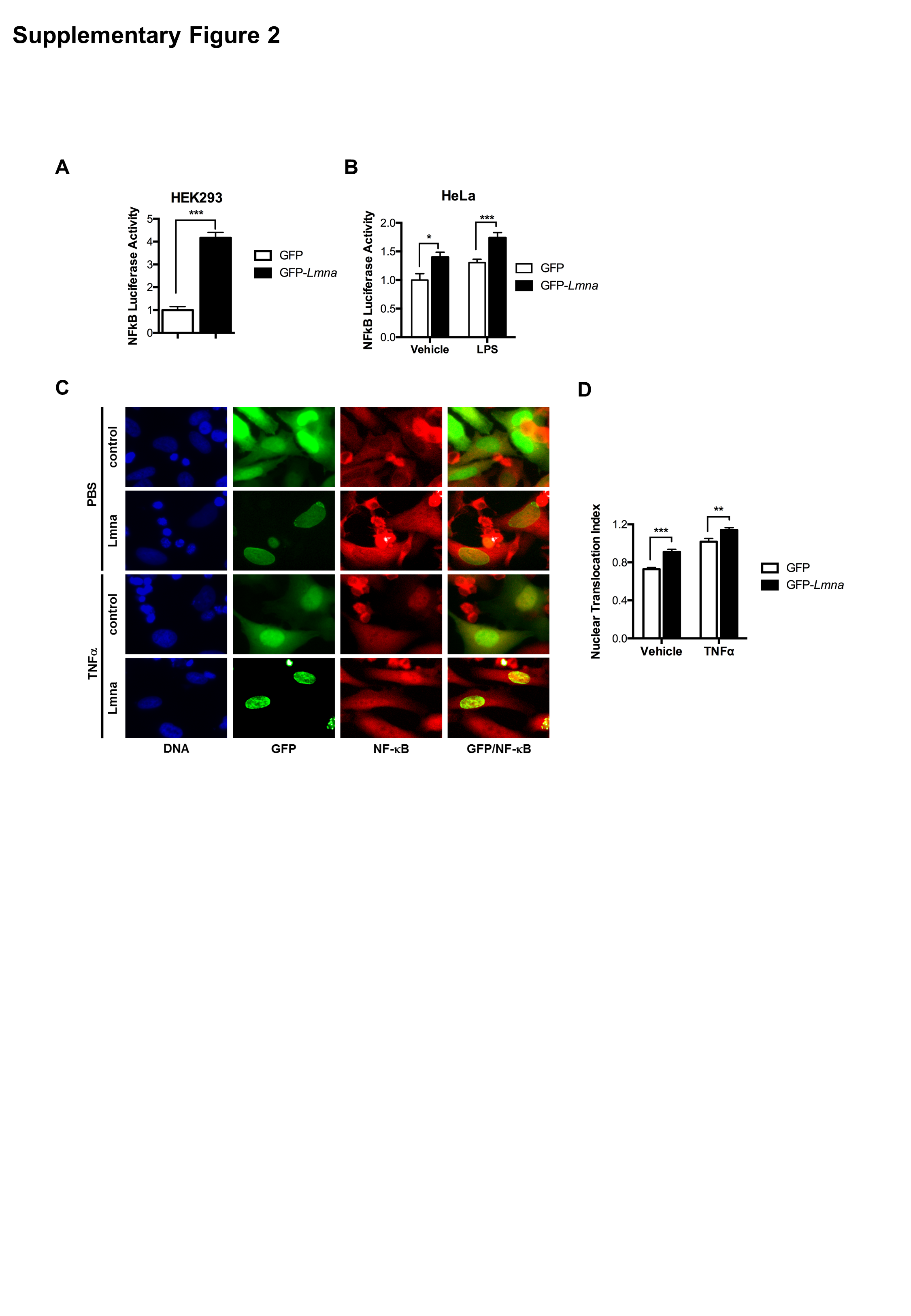

Supplement: Supplementary file 2 [file Image_2.TIF]
