# Supplementary material for: Macrophage Lamin A/C Regulates Inflammation and the Development of Obesity-Induced Insulin Resistance
Source: Front Immunol. 2018 Apr 20;9:696. doi: 10.3389/fimmu.2018.00696 (PMC5920030; doi:10.3389/fimmu.2018.00696)
Supplement: Supplementary file 3 [file Table_1.PDF]

Supplementary Material

Macrophage Lamin A/C Regulates Inflammatory Responses

Youngjo Kim[1], Princess Wendy Bayona[1], Miri Kim[1], Jiyeon Chang[1], Sunmin Hong[1], Yoona Park[1], Andrea Budiman[1], Young-Jin Kim[2], Chang Yong Choi[3], Woo Seok Kim[4], Jongsoon Lee[5,*], Kae Won Cho[1,*]

*** Correspondence:** Jongsoon Lee: jongsoon.lee@joslin.harvard.edu, Kae Won Cho: kwcho@sch.ac.kr

Supplementary Figure 1. Obesity increases the expression levels of lamin A/C in epididymal fat. Male C57BL/6 mice were fed normal chow diet (ND) or high-fat diet (HFD) for 12 weeks. qRT-PCR analysis of *Lmna*, *Lmnb1* and *Lmnb2* in eWAT, iWAT and liver from ND- and HFD-fed mice.

Supplementary Figure 2. Lamin A/C increase NF-κB activity *via* nuclear translocation of p65/Rel A in both basal and LPS-stimulated state. (A) NF-κB luciferase activity in HEK293 cells which were co-transfected with GFP or GFP-Lmna then detected by pNF-κB-Luc, a luciferase reporter construct that has κB responsible elements in the promoter. (B) NF-κB luciferase activity in HeLa cells co-transfected with GFP or GFP-Lmna together with pNF-κB-Luc. Luciferase activities were normalized by cells co-transfected with a non-reporter plasmid, pRL-SV40 overexpression. (C) Immunofluorescence images of HeLa cells transfected with GFP or GFP-*Lmna*. To induce inflammatory responses, transfected cells were treated with either PBS or TNFα. (D) Quantitation of nuclear translocation of p65/Rel A. To calculate the nuclear translocation index, average nuclear signal of p65/Rel A within a nucleus was divided by average cytoplasmic signal in the same cell. Error bars represent SEM. ** p <0.01, *** p <0.01.

Supplementary Table 1. Human subjects characteristics and metabolic parameters.

	Average ± S.D
Subjects (M/F)	30 (7/23)
Age, years	45.53 ± 12.8
BMI, kg/m^2	34.79 ± 9.2
Glucose, mg/dL	116.38 ± 25.70
HbA1c	10.03 ± 3.51
