# Supplementary material for: Macrophage Lamin A/C Regulates Inflammation and the Development of Obesity-Induced Insulin Resistance
Source: Front Immunol. 2018 Apr 20;9:696. doi: 10.3389/fimmu.2018.00696 (PMC5920030; doi:10.3389/fimmu.2018.00696)
Supplement: Supplementary file 4 [file Table_2.PDF]

Supplementary Table 2. Flow cytometry antibodies used in this study

Antibody	Clone	Company
CD64	X54-5/7.1	BD Pharmingen
CD11c	N418	eBioscience
CD45	30-F11	eBioscience
Lamin A/C	4A7	eBioscience
