# Supplementary material for: Macrophage Lamin A/C Regulates Inflammation and the Development of Obesity-Induced Insulin Resistance
Source: Front Immunol. 2018 Apr 20;9:696. doi: 10.3389/fimmu.2018.00696 (PMC5920030; doi:10.3389/fimmu.2018.00696)
Supplement: Supplementary file 5 [file Table_3.PDF]

Supplementary Table 3. Primer sequences for qRT-PCR in this study

Gene	Forward primer sequence (5'→3')	Reverse primer sequence (5'→3')
Lmna	AAGACCCTTGATTCTGTGGC	TCCAATGTGCGCTTCTCAC
Lmnb1	GCGGCACTAAACTCTAAGGATG	TTACGAAACTCCAAGTCCTCAG
Lmnb2	GTGGCATCAAGACCCTGTAC	GATTCCAGATCCTTCACTCGG
Il6	TAGTCCTTCCTACCCCAATTTCC	TTGGTCCTTAGCCACTCCTTC
TNF	CCCTCACACTCAGATCATCTTCT	GCTACGACGTGGGCTACAG
Ccl2	TTAAAAACCTGGATCGGAACCAA	GCATTAGCTTCAGATTTACGGGT
NFkB1	ATGGCAGACGATGATCCCTAC	TGTTGACAGTGGTATTTCTGGTG
Rela	AGGCTTCTGGGCCTTATGTG	TGCTTCTCTCGCCAGGAATAC
18S	TTGACGGAAGGGCACCACCAG	GCACCACCACCCACGGAATCG
